# Distinct venous thrombotic phenotypes exist in the critically ill

**DOI:** 10.62675/2965-2774.20260044

**Published:** 2026-03-16

**Authors:** Nicole P. Juffermans, Marieke J.H.A. Kruip, Henrik Endeman

**Affiliations:** 1 Erasmus Medical Center Department of Intensive Care Rotterdam Netherlands Department of Intensive Care, Erasmus Medical Center - Rotterdam, The Netherlands.; 2 Erasmus Medical Center The Emergency, Perioperative and Intensive Care Laboratory Rotterdam Netherlands The Emergency, Perioperative and Intensive Care Laboratory, Erasmus Medical Center - Rotterdam, the Netherlands.; 3 Erasmus University Medical Center Erasmus Medical Center Department of Hematology Rotterdam Netherlands Department of Hematology, Erasmus Medical Center, Erasmus University Medical Center - Rotterdam, the Netherlands.; 4 Erasmus University Medical Center Erasmus Medical Center Department of Quality and Patient Care Rotterdam Netherlands Department of Quality and Patient Care, Erasmus Medical Center, Erasmus University Medical Center - Rotterdam, the Netherlands.

**Keywords:** Venous thrombosis, Critical illness, Hypoxemia, Risk factors, Histology

## Abstract

In the critically ill, different thrombotic phenotypes exist. *In situ* thrombosis differs from the classical embolus. It is characterized by cellular infiltration along the vessel wall of the smaller pulmonary arteries, as well as by a dysregulated host immune response.

## INTRODUCTION

We have been thinking about venous thrombosis as a homogenous disease. We now know that for critically ill hypoxemic patients, this view is not true. This review challenges this old paradigm by describing different phenotypes of venous thrombosis. We also reflect on the potential consequences of this finding, in particular the question whether all thrombosis should be anticoagulated.

### High incidences of venous thrombosis in the critically ill

The incidence of venous thrombosis is high in pathologies such as viral pneumonia or cancer. Strikingly, these different pathologies have in common that the rate of thrombosis is always higher in patients who are critically ill when compared to ward patients. Thrombosis incidence can even reach up to 50% in specific critically ill patient populations, despite the use of thromboprophylaxis.^(1,2)^ This observation suggests that, besides the condition itself, disease severity drives the risk of thrombosis.

### Rethinking the venous thrombosis paradigm

In the critically ill, a high incidence of both pulmonary embolism (PE) and deep vein thrombosis (DVT) is found. These terms date back from 1859, when Virchow, upon examining corpses, suggested that "smaller fragments from a thrombus are carried along the current of blood and driven in remote vessels". Indeed, for a long time, we considered DVT and PE to be different manifestations of the same disease. We now know this is not always true.

It has been known for some time that in patients with PE, DVT cannot always be identified. Also, clinical prediction rules such as the Wells or the Padua criteria do not predict thrombosis in the critically ill. Collectively, this may suggest different pathophysiologic mechanisms. The coronavirus disease 2019 (COVID-19) pandemic, during which thrombi were "in our face", provided robust evidence, with post-mortem investigations revealing pulmonary thrombosis in the absence of DVT, indicating *de novo* or *in situ* thrombosis (IST).^(3)^ *In situ* thrombosis is attributed to endothelial dysfunction in combination with systemic inflammation, promoting *de novo* thrombus formation in pulmonary arteries.

We recently extended these findings^(4)^ again in patients who succumbed to COVID-19, by phenotyping IST from PE, using a combined analysis of histology, radiology, and immunology markers. We found that a layered fibrin structure histologically characterizes PE and is not attached to the vessel wall (Table 1). In line, computed tomography (CT) scans of PE show filling defects in the center of the pulmonary artery. In contrast, IST has a disorganized structure, with abundant macrophage and fibroblast infiltration, originating from the vascular wall. Radiologically, filling defects occur eccentrically along the vessel wall and are more often found in segmental and subsegmental arteries, in proximity to areas with parenchymal infiltrates. In addition, patients with IST consistently exhibited a hyperinflammatory immune response, with higher levels of interleukin (IL)-17, IL-18, and IL-33 than those with PE. While T-helper (Th) type 1 cell activation is crucial for adequate virus clearance, these cytokines indicate upregulation of Th2 and Th17 pathways, which are known to contribute to excessive host defense. This dysregulated response leads to activation of the local endothelium, which then loses its antithrombotic properties, promoting platelet activation and local aggregation.

**Table 1:** Characteristics of pulmonary embolism *versus* pulmonary in situ thrombosis

	Pulmonary embolism	In situ thrombosis
Histology	Layered structure not attached to the vessel wall	Disorganized structure originating from the vessel wall
Radiology	Central filling defect	Eccentric filling defect in more peripheral arteries in proximity to infiltrates
Immunology		Upregulation of markers of Th2 and Th17 pathways
Gas exchange	Impaired	Not known, may be variable

*In situ* thrombosis has also been described in other hyper-inflammatory conditions, such as antiphospholipid syndrome, trauma, and sepsis.^(5–7)^ Given the current absence of clear classifications, the default radiologic description of filling defects in the pulmonary arterial system is ‘pulmonary embolus’. Thereby, it is very likely that the incidence and morbidity caused by IST are significantly underestimated in the critically ill.

### The impact of different thrombotic phenotypes in the critically ill

The finding that IST is a different thrombotic phenotype than PE has several potential clinical implications.

First, for clarity, the nomenclature should be changed. As "pulmonary embolism" and "*in situ* thrombosis" can be clearly distinguished histologically, but radiological criteria are not defined and biomarkers are not clinically available, we propose transitioning to the default term "pulmonary thrombosis" instead of "pulmonary embolism".

**Figure 1:**
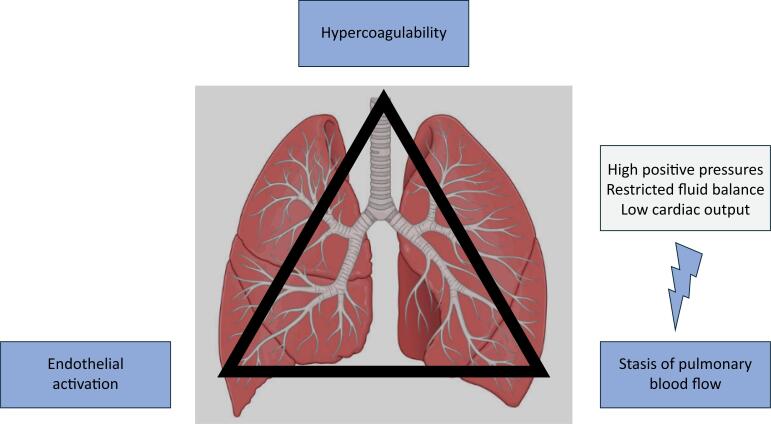
Virchow's triad for pulmonary thrombosis.

Second, different pathophysiology might implicate different risk factors. Indeed, as noted, classical risk scores do not apply in the intensive care unit (ICU). We again refer to Virchow, whose famous triad outlines three components of thrombosis (Figure 1). Severity of inflammation is a risk factor, driving both endothelial activation and systemic coagulopathy. It is not known which downstream effect of "inflammation" most strongly drives thrombosis, but hypoxemia may be an important factor. The third component of the triad is pulmonary blood flow, which has been largely ignored in studies on thrombosis in the critically ill but is highly relevant in daily ICU care. Mechanical ventilation with high positive pressures and restricted use of fluids can contribute to stasis of pulmonary blood flow. Also, modulating cardiac output will impact pulmonary blood flow. Given that these factors are to some extent modifiable in circulatory and ventilatory management, further insight into their contribution to the development of pulmonary thrombosis is warranted.

Third, identification of different pulmonary thrombosis phenotypes may have implications for anticoagulant management. Not all pulmonary thrombi may worsen gas exchange. In fact, when IST occurs in poorly ventilated areas due to infiltration, it may even improve the ventilation-perfusion (VQ) ratio. That said, however, some patients will have pulmonary thrombi that worsen gas exchange, and these need therapeutic anticoagulation. In addition, as IST is driven by inflammation, therapeutic anticoagulation may not be the most effective therapy. In line with this, intensifying anticoagulation does not improve outcome in critically ill COVID-19 patients.^(1,8)^ Alternatively, IST may respond better to anti-inflammatory treatments.

### How to proceed from here?

Most research was done on COVID-19. Thereby, whether findings apply to other inflammatory conditions is an important research question. To move forward, distinguishing PE from IST based on clinical markers is pivotal for understanding risk factors and pathophysiology and for guiding care decisions. Point-of-care diagnostics of non-Th1 pathway markers may be possible in the future. The utility of biomarkers more readily available in clinical practice, such as C-reactive protein or D-dimer, should be investigated. Also, radiologic identification of IST *versus* PE to provide a uniform distinction is an important goal, possibly aided by artificial intelligence. Once these characteristics are established, they can help stratify patients exposed to anticoagulant or anti-inflammatory therapies.

## CONCLUSION

In critically ill patients with hypoxemic respiratory failure and a hyper-inflammatory condition, *in situ* thrombosis and pulmonary embolism are different thrombotic phenotypes. *In situ* thrombosis is characterized on imaging by irregular filling defects along the walls of the smaller pulmonary arteries, in proximity to infiltrates, and by a dysregulated host response. This finding warrants investigation of risk factors and optimal management of *in situ* thrombosis.

## Data Availability

The contents underlying the research text are included in the manuscript.
